# Study of serum ctx in 50 oral surgical patients treated with oral bisphosphonates

**DOI:** 10.4317/medoral.17583

**Published:** 2011-12-06

**Authors:** Antonio J. Flichy-Fernández, Teresa Alegre-Domingo, Sandra González-Lemonnier, José Balaguer-Martínez, María Peñarrocha-Diago, Yolanda Jiménez-Soriano, David Peñarrocha-Diago, José V. Bagán-Sebastián

**Affiliations:** 1 DDS. Master in Oral Surgery and Implantology. Collaborating Professor of the Master in Oral Surgery and Implantology. Valencia University Medical and Dental School; 2 Associate Professor of Oral Surgery. Professor of the Master in Oral Surgery and Implantology. Valencia University Medical and Dental School; 3 Collaborating Professor in Oral Medicine. Valencia University Medical and Dental School; 4 Chairman of Stomatology. Valencia University Medical and Dental School. Head of the Service of Stomatology, Valencia University General Hospital. Valencia (Spain)

## Abstract

Objectives: To determine whether there is a relationship between the total BP dose administered and the variations in serum CTX concentration. Study design: The study included 50 patients requiring dental implant surgery and treated with oral BPs, seen in an Oral Surgery and Implantology Unit between January 2007 and June 2009. The patients were divided into two groups: those in which the medication was not suspended before obtaining the laboratory test sample, and those patients referred from other dental clinics in which BPs was suspended before reporting to our Unit. The total drug dosage administered and the total dose per kilogram body weight were evaluated for comparison with serum CTX. The data obtained were correlated to the osteonecrosis risk table developed by Marx et al. in 2007. Results: There were no significant differences between the two groups in relation to the total administered dose and the dose in mg/kg b.w. Likewise, in both groups no relationship was observed between the serum CTX value and the total administered dose or the dose in mg/kg b.w. No differences were found between the two patient groups regarding chemical osteonecrosis risk based on the criteria of Marx et al. Conclusions: No relationship was observed between the oral BP dose administered (total dose or expressed in mg/kg b.w.) and serum CTX concentration, and suspension of the medication did not influence the serum CTX levels.

** Key words:**Serum CTX, osteonecrosis, oral bisphosphonates.

## Introduction

Serum carboxyterminal cross-linking telopeptide of type I collagen (CTX) is a marker of osteoclast function. A number of authors have correlated serum CTX to the administration of bisphosphonates (BPs), such as Greenspan et al. (1), Chailurkit et al. (2) and Brown et al. (3), who reported a decrease in serum CTX following the administration of BPs. However, other authors such as Peris et al. (4) and Kunchur et al. (5) have observed no such relationship.

Marx et al. (6) reported a relationship between low serum CTX values and an increased incidence of osteonecrosis of the jaws (ONJ). These authors developed tables designed to establish the relative risk of developing ONJ as a function of the serum CTX levels. In this context, values under 100 pg/ml represented high risk, values between 100-150 pg/ml represented moderate risk, and concentrations of over 150 pg/ml were taken to indicate minimal risk. The investigators estimated that serum CTX increases between 25.9 - 26.4 pg/ml for each month without the administration of BPs.

The present study, involving a group of patients treated with BPs via the oral route, was designed to determine whether there is a relationship between the BP dose administered and the variations in serum CTX concentration.

## Material and Methods

-Patients

The study included 50 patients requiring dental implant surgery and treated with oral BPs, seen in the Oral Surgery and Implantology Unit of a University Dental Clinic between January 2007 and June 2009.

A case history was compiled on each patient, including age, gender, weight, disease antecedents of interest, and usual medication (drug, dose and duration of treatment). The specialists prescribing BP treatment were consulted to determine whether it would be possible to switch to some other type of medication.

In this context, two groups were established: group A (patients directly visiting our Unit and who continued to take BPs) and group B (patients who stopped taking BPs before collecting the laboratory test sample).

The patients in group B were subjects referred from private dental clinics to our Unit for treatment. Some of the dental surgeons referring the patients had already consulted the specialist about the possibility of suspending BP treatment, replacing it with some other type of drug. The rest of the referred patients stopped taking BPs on their own accord after being explained about the risk of complications, or because the prescribing specialist decided that BP treatment had concluded.

-Laboratory tests

The total drug dosage administered and the total dose per kilogram body weight were assessed for the study of serum CTX. The patients reported to the laboratory under fasting conditions. The data obtained were correlated to the osteonecrosis risk table developed by Marx et al. in 2007.

-Statistical analysis

A descriptive analysis was made in both patient groups of gender, age, body weight, medication, total dosage and dose expressed as mg/kg body weight, data accuracy and serum CTX concentration. Sample distribution was assessed, and in those cases where a non-normal distribution was suspected, we applied the corresponding nonparametric tests, with the same results. The statistical analysis was performed using the SPSS version 15.0 statistical package for Microsoft Windows (SPSS Inc., Chicago, IL, USA).

## Results

Two patients refused laboratory testing and were excluded from the study. A total of 50 patients were evaluated: one male and 49 females. The mean patient age was 63.8 years (range 51-77). The mean body weight was 60.6 kg (range 44-79). Patient distribution according to the administered BP is shown in ( Table 1).

**Table 1:**
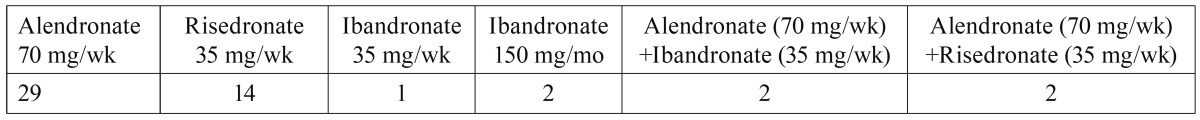
Patient distribution according to the drug prescribed.

Eighty percent of the patients knew the exact timing of their medication (with a report from the prescribing physician, stating the exact date on which treatment was started), while 20% were only able to offer an estimate (without the corresponding report from the prescribing physician). The mean duration of BP therapy was 42 months (range 4-132). As regards patient distribution, 46% belonged to group A and 54% to group B. In the latter group, the mean time without BP treatment was 5.8 months (range 1-12).

The serum CTX concentration ranged between 60-880 pg/ml. On classifying the laboratory test data according to the ranges described by Marx et al. (6) for assessing the risk of bone necrosis, 22% were seen to present no risk (≥ 300 pg/ml), 44% minimum risk (150-299 pg/ml), 22% moderate risk (100-150 pg/ml) and 12% high risk (˂100 pg/ml).

**Figure 1:**
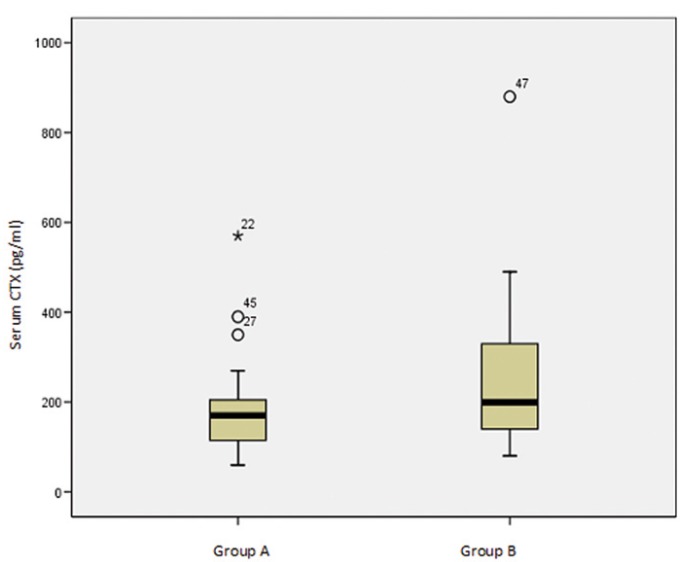
Relationship between the two study groups in reference to serum CTX concentration.

There were no statistically significant differences between the two groups (p>0.005) regarding the total administered dose (F=2.095), the total dose per kg body weight (F=0.778), the serum CTX values (F=1.697) (Fig. 1), or osteonecrosis risk (χ2=2.972) (according to the criteria of Marx et al. 2007).

**Figure 2:**
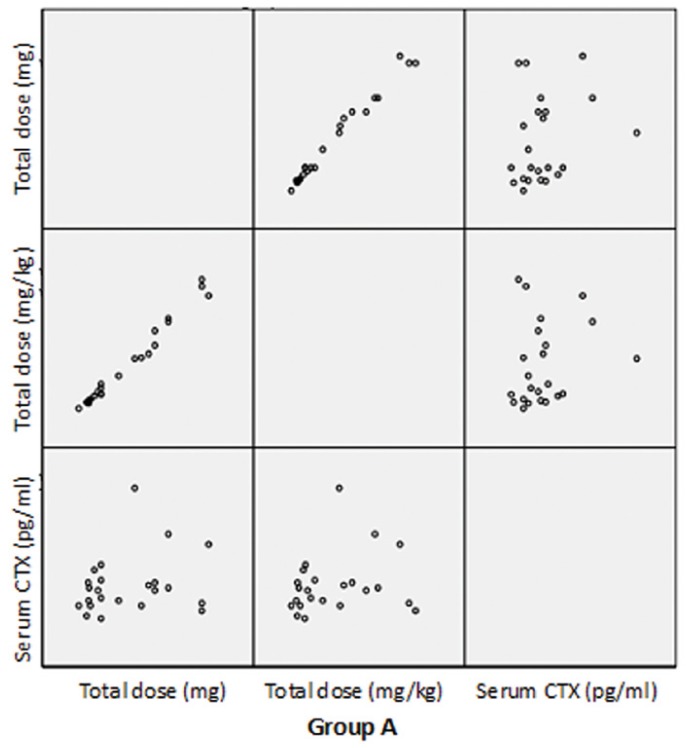
Dispersion chart showing the relationship between the administered bisphosphonate doses and serum CTX concentration in the two study groups.

**Figure 3:**
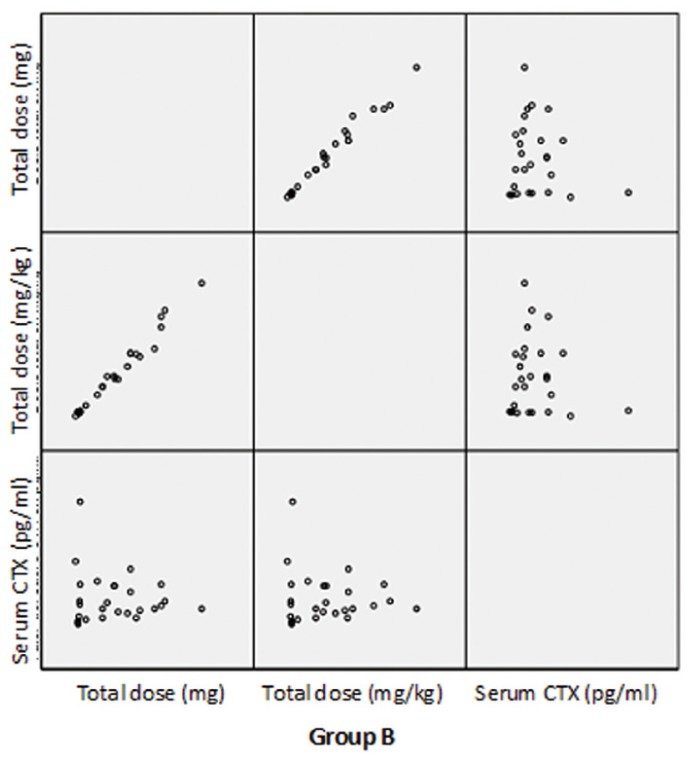
Dispersion chart showing the relationship between the administered bisphosphonate doses and serum CTX concentration in the two study groups.

The dispersion chart in figures 2, 3 shows that no relationship was found between the total drug dosage administered or the total dose per kilogram body weight and the serum CTX concentration.

## Discussion

A number of studies have related treatment with oral bisphosphonates (BPs) to reductions in bone resorption markers. Chailurkit et al. (2) administered 10 mg of alendronate a day to 70 patients, and recorded a significant reduction in serum CTX after both 6 months and one year of treatment. Brown et al. (3), in 125 patients with bone metastases, reported a significant reduction in serum CTX at daily doses of ≥ 1600 mg of clodronate via the oral route, after 6 months of treatment. In the present study, the mean duration of treatment was 42 months, which is significantly longer than the duration of therapy in the studies of Chailurkit et al. (2) and Brown et al. (3).

Peris et al. (4), in patients with Paget’s disease, recorded no significant decrease in serum CTX following treatment with BPs. In our series we likewise found no relationship between the total drug dosage administered or the total dose per kilogram body weight and the serum CTX concentration.

Marx et al. (6), in patients with bone necrosis secondary to BP treatment, found a relationship between osteonecrosis risk and serum CTX. In contrast to the results obtained by Marx et al. (6), in our study there was no relationship between serum CTX and the risk of bone necrosis.

In turn, Kunchur et al. (5) evaluated the efficacy of serum CTX testing in assessing the risk of osteonecrosis in 348 patients treated with oral BPs. According to these authors, the mentioned serum test is unable to predict bone necrosis risk, in coincidence with our own findings.

A number of studies involving patients receiving BPs via the intravenous route likewise reported no relationship between serum CTX and the risk of bone necrosis. This is the case for example of the articles published by Lehrer et al. (7) and Bagán et al. (8), who recorded no correlation between serum CTX and exposed areas of bone necrosis.

On the basis of the total drug dose, the duration of treatment, patient body weight and the time of treatment suspension before the study, we conclude that it is doubtful whether the serum CTX test is of help in determining osteonecrosis risk in patients treated with oral bisphosphonates. Nevertheless, studies involving larger sample sizes are needed to confirm these findings.
